# Undernutrition and associated factors among incarcerated people in Mizan prison institute, southwest Ethiopia

**DOI:** 10.1371/journal.pone.0251364

**Published:** 2021-05-11

**Authors:** Wondimagegn Wondimu, Bethlehem Girma, Melese Sinaga, Abonesh Taye

**Affiliations:** 1 Department Epidemiology, School of Public Health, College of Medicine and Health Sciences, Mizan Tepi University, Mizan Aman, Ethiopia; 2 Department of Health Extension Program, Mizan Aman College of Health Sciences, Mizan Aman, Ethiopia; 3 Department of Nutrition and Dietetics, Faculty of Public Health, Jimma University, Jimma, Ethiopia; University of Mississippi Medical Center, UNITED STATES

## Abstract

**Background:**

In resource-limited countries like Ethiopia, where malnutrition is a common problem, incarcerated people’s sentences might be changed into a death sentence if the problems of undernutrition are not well understood and managed properly. There is limited evidence on nutritional status and associated factors among incarcerated people in low- income countries like Ethiopia, including the study area.

**Objective:**

To assess the magnitude of undernutrition and associated factors among incarcerated people in Mizan prison institute, southwest Ethiopia.

**Methods:**

An institution based cross-sectional study was conducted among 340 incarcerated people in Mizan prison institute from April 1 to 27, 2020, using a stratified sampling technique. An interviewer-administered structured questionnaire was used to collect data. The outcome variable (undernutrition) was assessed by measuring body mass index (BMI). Binary logistic regression was used to identify factors associated with undernutrition. Adjusted odds ratio (AOR) and 95% confidence intervals (CI) were used to measure the strength of association and a p-value less than 0.05 was used to declare the level of statistical significance.

**Results:**

The magnitude of undernutrition was 18.6% (95%CI: 14.4%, 22.8%). Being in the age category of 18–29 years (AOR = 2.60; 95%CI: 1.22,5.52), history of previous incarceration (AOR = 2.31;95%CI: 1.23,4.34), duration of imprisonment (AOR = 1.19; 95%CI: 1.05,1.34), having depression (AOR = 2.1; 95% CI: 1.10,3.97) and sleeping in group (AOR = 2.17; 95% CI: 1.18,4.01) were factors significantly associated with an increased odds of undernutrition. However, the presence of family support significantly decreased (AOR = 0.29; 95%CI: 0.12, 0.69) the odds of undernutrition.

**Conclusion:**

The magnitude of undernutrition in the prison was found to be comparable to that of the general population in Ethiopia. The efforts on the ground to tackle undernutrition in the general population shall be extended to incarcerated people, especially by focusing on vulnerable groups such as those who had longer durations of incarceration, history of previous imprisonment, depression and no support.

## Introduction

Incarcerated people, as human beings, have the right to a standard of living adequate for health, including food [1]. However, evidence shows that the basic human rights of incarcerated people are not sufficiently respected [2–4]. Prisons contain a high number of people with serious and often life-threatening conditions [5]. Nutrition related abnormalities are among the health problems that incarcerated people suffer from. These include micronutrient deficiencies, delayed recovery and increased risk of mortality [2, 6]. Undernutrition, which is common among incarcerated people, refers to an insufficient intake of energy and nutrients to meet an individual’s needs to maintain good health. It is not only the problem of the quantity of the food but also the quality [6, 7]. People incarcerated in developing countries have been shown to be vulnerable to different dietary deficiency diseases [8]. Although there is limited evidence on the nutritional problems of incarcerated people, available studies from abroad revealed that the prevalence of undernutrition among incarcerated people ranged from 14.06% to 39.7% [9–13]. The magnitude of undernutrition among incarcerated people with HIV/AIDS in Addis Ababa, Ethiopia was 43% and among those with respiratory tract infection in Tigray was 25.2% [2, 14].

Nutritional problems in prison can result in severe adverse outcomes. Nutritional inadequacy places incarcerated people at a higher risk of developing acute and chronic nutritional deficiency diseases [15]. As a result, incarcerated people will return to the community, carrying back with them new diseases and untreated conditions that may pose a threat to community health and add to the burden of disease in the community. In addition, incarcerated people’s sentences might be changed into death sentences if the problems of undernutrition are not understood well and managed properly, in resource-limited countries like Ethiopia where malnutrition problems are very common [2, 16].

In Ethiopia, incarcerated people get their food mainly from the prison institution. Food in Ethiopian prisons is insufficient both in quality and quantity for the incarcerated people, and also there are no rules in state or federal laws or policies on nutritional standards of food [17]. The main dish in all the prisons is injera (local bread) and stew, which is most of the time made with beans, usually with no meat. Incarcerated people are fed three times a day with the same meal [18].

Based on previous studies done in prison setting the factors that were identified as the significant determinants of undernutrition among incarcerated people include old age, male sex, history of previous incarceration, long duration of incarceration, depression, taking two meals a day instead of three, absence of family visits and lack of financial assistance from family [2, 13, 14, 19].

The World Prison Brief (WPB) and Institute for Criminal Policy Research (ICPR) reported that the total prison population in Ethiopia increased from 85 per 100,000 total populations in 2000 to 127 per 100,000 in 2014 [20]. Despite this high number of incarcerated people and the public health importance of the problem, undernutrition among incarcerated people is inadequately understood in Ethiopia. To the authors’ best knowledge, this is the first study to assess undernutrition and associated factors among a representative sample of incarcerated people within an Ethiopian prison. Two previous studies had been conducted in Ethiopia which assessed undernutrition on incarcerated people with HIV/AIDS and respiratory tract infections, respectively [2, 14]. However, these studies were not inclusive of the entire prison population.

As a result, this study aimed to assess undernutrition and associated factors among incarcerated people in Mizan prison institute, southwest Ethiopia.

## Methods

### Study setting, design, and population

A cross-sectional study was conducted at Mizan prison institute, which is located in Mizan town. Mizan town is the capital of the Bench Sheko zone, which is found in 585 km away from Addis Ababa, the capital of Ethiopia, in the southwest direction. The prison was established in 1951 and it is the only prison institute in Bench Sheko zone. There was one clinic in the prison with one health officer, four nurses, one pharmacist and one laboratory technician during data collection. The prison had also one primary school and six food and drink establishments which were serving the incarcerated people. In the food and drink establishments mainly bread, fruits, vegetables, dairy products, soft drinks and hot drinks such as coffee and tea are available for the incarcerated people who can afford the servings. At the time of data collection, there were 2,061 incarcerated people where, 2,005 were males and 56 were females. The study was conducted from April 1 to 27, 2020. All incarcerated people who were 18 years old and above, and stayed in the prison for at least 6 months were included in the study. Trusted incarcerated people and those who were not able to respond due to different medical-related conditions were excluded from the study. Trusted incarcerated people are those with good discipline and believed not to commit any further crime. They are allowed to move out of the prison compound which enables them to address their different needs such as food, and they are also responsible to control other incarcerated people.

### Sample size and sampling procedure

The sample size was determined by using a single population proportion formula assuming a 95% confidence level and 5% margin of error. In addition, the proportion of undernutrition among incarcerated people was taken as 50%. By adding a non-response rate of 5% and using reduction formula (since the population was finite) finally, the working sample size was 339.99 ≈ **340**.

A stratified sampling technique followed by simple random sampling was used to select the study participants. First, the sampling frame was created by registering all eligible incarcerated people found in each cell of the prison. Then after stratifying the sampling frame by sex a unique number was given for each eligible participant and the sample size was proportionally allocated to the size of each stratum, then finally computer-generated random number selection technique was used to select the study participants.

### Data collection and measurement of the variables

Data were collected by using a structured interviewer’s administered questionnaire which was adapted from related literatures [21–23]. The questionnaire was translated into Amharic language and back-translated to English by language experts to keep the consistency of the questions.

The questionnaire was pretested on 5% of the study population in Bonga prison institute which is 136 km away from Mizan prison institute and some modifications of items (such as wording and order of questions) were made. The questionnaire contains questions on socio-demographic, prison–related, medical, nutrition-related and behavioral characteristics. Four trained BSc. holder nurses collected the data with the supervision of the principal investigators. The Pearson correlation coefficient (r) was used to assess the internal validity of our tool and the computation revealed that our tool was valid enough. i.e. the minimum calculated r was 0.203 which was significantly (p = 0.001) higher than the critical value (0.107) with degree of freedom (df) = 332 and two-sided α = 0.05.

#### Anthropometric measurements

The weight and height of the participants were measured using a digital standing weight scale and stadiometer (Detecto, UK) which measures weight and height together. The weight scale was calibrated to zero before measuring each participant and the accuracy of the instrument was checked by measuring the weight of a known object. The accuracy of the stadiometer was also checked by measuring the height of an object with known height. Both measurements were done in light clothing, bare feet and no headwear. Weight was recorded to the nearest 0.1 kg.

Height was measured by using the stadiometer while the respondents were standing erect against the stadiometer with the shoulder, buttock, calf and heels touching the stadiometer, eyes looking straight ahead (Frankfurt plane) so that the line of sight was perpendicular to the body. The values were recorded to the nearest 0.1 cm.

#### Dietary diversity score

Individual Dietary Diversity Score (IDDS) was measured after dietary data were collected using a 24-hour dietary recall method. Any type of food that was consumed within 24hrs just before the time of data collection was recorded. Foods eaten by the respondents were classified into 10 food groups: all starchy staples, beans and peas, nuts and seeds, all dairy, flesh foods, eggs, vitamin A-rich dark green leafy vegetables, other vitamin A-rich vegetables and fruits, other vegetables, other fruits.

Participants received 1 point if they consumed a minimum of one food within each subgroup within the past 24 hours, and 0 points if they did not. Points were summed up to get the total IDDS score. Participants who had a score of at least 5 were classified as having a diversified diet; otherwise their diet was considered non-diversified. [21, 24].

#### Physical activity

The physical activity of the participants was measured by adapting the global physical activity questionnaire (GPAQ) considering physical activity done in work and recreational activities. The activities were classified as moderate-intensity and vigorous intensity. The total time spent in physical activity during a typical week (i.e. duration of physical activity in minutes and frequency of physical activity per week) and intensity of the physical activity was multiplied by four if the intensity was moderate and by eight if the intensity was vigorous. Total activity was summed and dichotomized into physically active (600+ MET minutes/week) and physically inactive (<600MET/week). MET is the ratio of a person’s working metabolic rate relative to the resting metabolic rate. One MET is defined as the energy cost of sitting quietly, and is equivalent to a caloric consumption of 1kcal/kg/hour [25].

#### Depression

Patient Health Questionnaire (PHQ-9), a nine-item version was used to assess depression. Each depressive symptom on PHQ-9 was rated on a scale ranging from zero (not at all) to three (nearly every day). Depression total scores were computed for every one of the participants by adding scores of all the nine items of the scale. A participant was considered to be in the state of depression if he/she scored five and above [19, 23].

#### Alcohol consumption

Alcohol consumption was measured using the concept of a standard drink. Self-reported alcohol consumption was assessed via quantity-frequency (number of standard drinks consumed in a typical day x number of days in a week) method based on the Nordic Alcohol Consumption Inventory from which the average weekly alcohol consumption was calculated. Drinkers were those who drunk more than 1 unit (10 grams) of alcohol per week, before they were incarcerated. Similarly non-drinkers were those who drunk 1 unit (10 grams) or less alcohol per week, before they were incarcerated [22, 26].

#### Chat chewing

Chat chewing was measured considering both life time chewing duration (measured in year) and time spent in a single chewing session (measured in hour). Participants who used chat for more than five years and chewed for more than four hours in a single chewing session before they were incarcerated were considered as chat chewers [27].

#### Cigarette smoking

Participants who had smoked at least 100 cigarettes in his or her lifetime before they were incarcerated were classified as smokers [28].

### Data analysis

Data were checked for completeness and consistency during data collection and the responses in each question were coded for simplicity of data entry. The data were entered by using the Epi-data manager version 4.0.2.101 and exported to SPSS version 21 for cleaning and analysis. Descriptive analysis was done to explore the range of values, identify missing data or possibly miscoded data. Then, an exploratory data analysis was performed for the distribution of age and body mass index (BMI) so that the appropriate measure of central tendency could be used. The distribution of age and BMI were skewed to the right with the p-value of the Kolmogorov-Smirnov test result less than 0.05 indicating that the distributions were not normal. So, depending on their distribution median with its respective interquartile range was used to summarize these variables. The outcome variable was undernutrition and assessed by measuring BMI. A cutoff point of BMI less than 18.5 kg/m^2^ was used to define undernutrition. The explanatory variables include socio-demographic characteristics (age, sex, residence, religion, marital status, educational status, previous occupation, and support from family or other), nutrition related factors (dietary diversity, meal frequency and additional food), prison related factors (duration of imprisonment, history of previous imprisonment, and job in the prison), medical factors (depression, history of TB, history of malaria, history of diarrheal disease and history of HIV testing and its result) and behavioral factors (way of sleeping, physical activity, history of alcohol drinking, history of chat chewing and history of smoking). A binary logistic regression was used to analyze the association between the outcome variable (undernutrition) and the independent variables (factors). In bi-variable logistic regression, those variables with p-value <0.25 were eligible for multivariable analysis. The odds ratio (OR) with the respective 95% confidence interval was used to measure the strength of association. The final significance was decided using a p-value < 0.05. Multicollinearity was checked by checking the variance inflation factor (VIF) and no multicollinearity was found. The maximum standard error and VIF were 1.082 and 1.4 respectively. The goodness of fit (GOF) of the model was checked using Hosmer and Lemeshow GOF test. The p-value of the Hosmer and Lemeshow GOF test of the model was 0.631 which indicated that the model was good fit.

### Data quality management

To ensure the quality of the data the following measures were taken. Two days training was given for data collectors regarding the purpose of the study, measuring of weight and height, the confidentiality of the information that was collected and other ethical issues. Data were checked for completeness and consistency daily by the principal investigators and the necessary correction was done. The measuring scales were regularly tested and calibrated before each measurement by using objects with known weight and height measurements.

### Ethical consideration

The study proposal was approved by the Institutional Review Board (IRB) of the institute of health in Jimma University and approval letter with reference number IRB0006/2020 was obtained. The study participants were briefed about the purpose of the study, their right to refuse or discontinue participation, at any point of time during data collection and also confidentiality was ensured. Each participant was clearly informed in advance that their participation would not be considered in decisions regarding his /her release or future detention. Participation was completely voluntary, with no economic or other motivational incentive and a written informed consent was taken from each participant. The possible prevention methods of Corona Virus Disease 19 (COVID-19) were implemented during data collection. The collected data were kept in secured places and the name of the participants was not collected. After data collection, those participants who were under the category of undernutrition and /or had any level of depression were linked to the prison clinic so that the case could be managed accordingly.

## Results

### Socio demographic and prison-related characteristics of the participants

Among the total sample of 340, 334 were included in this study for the response rate of 98.2%. The majority of the study participants were males (95.2%), the median [±inter quartile range (IQR)] age of the respondents was 29 (±18.25) years and nearly half (47.3%) of the respondents were in the age group of 18–29 years. More than half (54.8%) of the participants attended primary education and 70.7% had a rural residence. Among the total participants, only one fourth (24.6%) get additional support in terms of food, money, soap and cloth from their families or others. About two thirds (64.7%) of the respondents stayed in prison for more than one year and about one fourth (23.4%) were imprisoned previously. Only 14.1% of them had a job in the prison (Table 1).

**Table 1:** Socio demographic and prison-related characteristics of incarcerated people in Mizan prison institute, southwest Ethiopia, 2020 (n = 334).

Variable	Category	Frequency (n)	Percent (%)
**Sex**	Male	318	95.2
	Female	16	4.8
**Age**	18–29	158	47.3
	30–39	72	21.5
	≥ 40	104	31.2
**Previous residence**	Rural	236	70.7
	Urban	98	29.3
**Religion**	Protestant	248	74.3
	Orthodox Tewahido	63	18.9
	Muslim	7	2.1
	Others ^a^	16	4.8
**Marital status**	Single	101	30.2
	Married	214	64.1
	Others ^b^	19	5.7
**Educational status**	never attended school and unable to read/write	70	20.9
	never attended school and able to read/write	17	5.1
	Attended/completed primary school (1–8)	183	54.8
	Attended/completed secondary school or above	64	19.2
**Previous occupation**	Farmer	208	62.3
	Private/government employee	47	14.1
	Other ^c^	79	23.6
**Support from family or others**	Yes	82	24.6
	No	252	75.4
**Type of support** *	Food	51	15. 3
	Financial support	70	21
	Other ^d^	6	1.8
**Duration of imprisonment**	< 12 month	118	35.3
	≥ 12 month	216	64.7
**Imprisoned previously**	Yes	78	23.4
	No	256	76.6
**Job in prison**	Yes	47	14.1
	No	287	85.9

a-Catholic& traditional beliefs

b-Divorced, separated & widowed

c–broker, housewife, student

d–cloth & soap

*a participant can have more than one support at a time.

### Medical, behavioral and environmental characteristics of the participants

Out of all respondents only 2.7% participants were diagnosed and treated for tuberculosis (TB) in the past 12 months. Out of one hundred thirty (38.9%) respondents who were ever tested for human immune virus (HIV), 6.2% were positive. Similarly, 29.9% respondents had depression. More than three fourths (76. 3%) of the respondents were physically inactive and 43.7% of the participants sleep in group (i.e. two or more incarcerated people sleeping on a single mattress) (Table 2).

**Table 2:** Medical, behavioral and environmental characteristics of incarcerated people in Mizan prison institute, southwest Ethiopia, 2020 (n = 334).

Variables	Frequency	Percent
**Had TB in the last 12 months**	9	2.7
**Had malaria in the last 2 weeks**	54	16.2
**Had diarrhea in the last 2 weeks**	78	23.4
**Ever tested for HIV**	130	38.9
**Positive HIV test result**	8	6.2
**Had depression**	100	29.9
**Physically active**	79	23.7
**Sleeping in group**	146	43.7
**Had history of smoking**	38	11.4
**Had history of alcohol drinking**	144	43.1
**Had history of chat chewing**	15	4.5

### Nutritional characteristics of the participants

Only 22.5% of the participants consumed diversified diet whereas more than three fourths (77.5%) consumed that of non-diversified. Similarly, only 22.2% participants got additional food other than the food that was given by the prison. Regarding the meal frequency, 3%, 35%, 56.3% and 15.7% of the respondents ate one, two, three and four or more times per day respectively. The median body mass index (BMI) of the participants was 21.49 (IQR ± 3.41). Among the total participants 18.6% (95%CI: 14.4%, 22.8%) were undernourished (BMI < 18.5 kg/m^2)^, from which 3% had mild undernutrition (17–18.4 kg/m^2^) and 15.6% had moderate (16–16.9 kg/m^2^) undernutrition. None of the study participants had severe undernutrition (< 16 kg/m^2^)^.^

### Factors associated with undernutrition

In the bi-variable analysis the following variables were found to be a candidate (p<0.25) for the multivariable logistic regression; age, previous occupation, history of the previous imprisonment, getting additional food, getting support, sleeping condition, depression, duration of imprisonment and experiencing diarrhea in the past two weeks.

On a multivariable logistic regression model, six factors including age group, duration of imprisonment, previous history of imprisonment, presence of support, depression and sleeping condition were significantly associated (P<0.05) with undernutrition. Participants who were depressed had two times [AOR = 2.1; 95% CI: (1.10, 3.97)] higher odds of undernutrition compared to those who were not depressed. Similarly, those who sleep in group had two times [AOR = 2.17; 95% CI: (1.18, 4.01)] higher odds of undernutrition compared to those who sleep alone. Moreover, incarcerated people in the age group of 18–29 had two and half times [AOR = 2. 60; 95%CI: (1.22, 5.52)] higher odds of undernutrition compared to those in the age group > 40. Similarly, those who were imprisoned previously had more than two times [AOR = 2.31; 95%CI: (1.23, 4.34)] higher odds of undernutrition compared to those who had no history of previous imprisonment. It was also observed that a one-year increase in incarceration leads to 19% [AOR = 1.19; 95%CI: (1.05–1.34)] higher odds of undernutrition. On the other hand, those who had support from family or others had 71% [AOR = 0. 29; 95%CI: (0.12, 0.69)] reduced odds of becoming undernourished than those who get no support (Table 3).

**Table 3:** Multivariable logistic regression model for factors associated with undernutrition among incarcerated people in Mizan prison institute, southwest Ethiopia, 2020 (n = 334).

Variables	Variables’ Category	Undernutrition (n = 334)		COR (95%CI)	AOR (95%CI)
		Yes	No		
**Age (years)**	18–29	37	131	1.69 (0.86–3.32)	2.60 (1.22–5.52)*
	30–39	11	57	1.16 (0.49–2.73)	1.50 (0.59–3.78)
	≥40	14	84	1	1
**Occupation**	Farmer	39	169	0.84 (0.44–1.59)	0.72 (0.34–1.55)
	Private/ Gov’t employee	6	41	0.53 (0.19–1.47)	0.92 (0.30–2.84)
	Other	17	62	1	1
**Support**	Yes	7	75	0.33 (0.15–0.77)	0.29 (0.12–0.69)*
	No	55	197	1	1
Duration of imprisonment in years**	-	62	271	1.17(1.05–1.30)	1.19 (1.05–1.34)*
**Imprisoned previously**	Yes	25	53	2.79 (1.55–5.03)	2.31 (1.23–4.34)*
	No	37	219	1	1
**Additional food**	Yes	10	64	1	0.97 (0.0.40–2.33)
	No	52	208	1.60 (0.77–3.33)	1
**Diarrhea in the last 2 weeks**	Yes	18	60	1.45(0.78–2.68)	1.62 (0.81–3.23)
	No	44	212	1	1
**Depression**	Depressed	27	73	2.10 (1.19–3.72)	2.10 (1.10–3.97)*
	Not depressed	35	199	1	1
	**Sleeping condition**	In group	36	110	2.04(1.17–3.57)	2.17 (1.18–4.01)*
Individually		26	162	1	1

*-significant at p-value less than 0.05.

**-this variable was treated as continuous. Thus, has no categories.

## Discussion

The finding from this study revealed that 18.6% (95%CI: 14.4%, 22.8%) of the respondents were undernourished. This prevalence was lower than the findings obtained from studies in Kality (43%) [14] and Tigray region prisons (25.2%) [2]. This discrepancy may be due to the difference in the study participants; the study participants in Kality and Tigray region had additional co-morbidities. i.e. they were HIV infected and had upper respiratory tract infection respectively [2, 14]. However, the prevalence was higher than the findings of the studies conducted in Iran (14.2%) and Côte d’Ivoire (14.06%) [11, 12]. This discrepancy may be due to the difference in socioeconomic statuses in the study areas (i.e. Both Iran and Côte d’Ivoire have relatively better statuses; for instance, in terms of health services). The current prevalence of undernutrition is comparable to that of the estimated prevalence of undernutrition in the general population of Ethiopia (19%) [29].

This study shows that the odds of undernutrition was two and half times higher among incarcerated people whose age ranged from18 to 29 compared to those aged 40 and above. The finding was unsupported by the study report from Kality prison in which the older age group had higher odds of undernutrition [14]. The discrepancy might be attributable to the differences in the health status of the study participants. The higher odds of undernutrition among the younger participants might be due to that younger incarcerated people are more likely to be affected by depression which is also significantly associated with undernutrition in this study. In addition, older individuals can cope with depression better than younger ones [30, 31]. Another explanation could be that the higher probability of young adults’ engagement in substance use, which is the recognized risk factor for undernutrition [32].

This study reveals that a one-year increase in incarceration leads to a 19% higher odds of undernutrition. This is in line with the finding of a study done in the Tigray region’s prisons where the odds of undernutrition increased by 7% for every year increment in incarceration [2]. Another study done in Antanimora females’ prison also showed that undernourished women had longer incarceration durations than well-nourished women [13]. This might be due to that, remaining in prison for a longer period of time increases the exposure of the incarcerated people to the harsh living environment such as poor environmental sanitation, lack of personal hygiene, psychological stress and infections which in turn lead to undernutrition [2].

The participants who were imprisoned previously had more than two times higher odds of undernutrition compared to those who had no history of the previous imprisonment. This finding is supported by the finding of the study done in Tigray [2]. The possible explanation could be that individuals with the history of incarceration had an increased probability of having multiple medical problems which could predispose them to undernutrition [33]. Another reason might be that employment rate and earning of individuals released from prison is low and had an increased probability of recidivism and undernutrition [34]. Moreover, undernutrition by itself could lead to different criminal behaviors and make an individual be imprisoned repeatedly [35].

This study shows that participants who had support from family or others had 71% reduced odds of becoming undernourished than those who got no support and this is comparable with the finding of a study done in northern Ethiopia where there was 41% reduction in odds of undernutrition among incarcerated people who had support [2]. This might be due to the additional access to adequate and diversified food that this affords them. Incarcerated people can get diversified food if the prison food menu is improved. This can be achieved by initiating rehabilitated prison farm system; i.e. nutrition sensitive agricultural activities in the prison which can be implemented by the collaboration of prison institution, incarcerated people and agricultural sectors. This intervention not only improves the incarcerated people’s daily ration, but also the income of incarcerated people particularly those who had no support [36]. Evidence also supports that psychosocial assistance had a positive effect on nutritional status either to treat undernutrition or maintain normal nutritional status in developing nations [37, 38].

Participants who were depressed were two times more likely to be undernourished than those who were not depressed. This could be due to loss of appetite, which often accompanies depression, and may lead to undernutrition [39]. Another explanation could be that, most of the time incarcerated people are cooperative within their group and they may share what they have including food but the motivational and affective profile associated with depression could influence the ability of depressed respondents to socially interact [40, 41]. Therefore, arranging psychological supports and recreational activities in the prison should be considered to address the depression among incarcerated people. Mental health services can be provided in the prison clinics by availing necessary resources. Incarcerated people should also be encouraged to do physical activities which can improve their mental health [42]. These interventions need resources and the commitment of the prison institution.

In this study, respondents who sleep in group had two times higher odds of becoming undernourished than those who sleep alone. Although there was no evidence that show the direct association between the sleeping condition and the nutritional status of incarcerated people, there could be indirect. Findings from elsewhere revealed that, there was a higher risk of intestinal tract infection among incarcerated people who sleep in group compared to those who had a separate bed [43]. Similarly, sleeping in a group signals the problem of overcrowding which is common in prisons and risk factors for many health problems like TB [44]. Both intestinal infection and TB are the known predisposing factors for undernutrition [45, 46]. Thus, preparing adequate living rooms and sleeping beds can reduce the problems of overcrowding and in turn that of undernutrition and many communicable diseases.

### Limitation of the study

The limitation of this study is that it used cross sectional study design in which one can’t determine the direction of association. The second limitation is that since pre-incarceration BMI was not known, it was difficult to determine whether imprisonment caused a change in BMI. Furthermore, it is possible that our results are subject to recall and social desirability bias. Finally, when assessing undernutrition, our study relied on BMI which is not sensitive enough to recognize small yet clinically significant weight loss [47].

## Conclusion

The overall magnitude of undernutrition among incarcerated people in the Mizan prison institute was found to be comparable to that of the general population in Ethiopia. Special attention should be given to dietary intake of incarcerated people, particularly focusing on those who are younger or had previous history of imprisonment, longer duration of incarceration, depression, and no outside support. The prison institute should arrange appropriate sleeping places for every incarcerated person so that they can sleep separately and there will be a decreased risk of communicable diseases which will in turn decrease the risk of undernutrition. Moreover, diversified food shall be provided for the incarcerated people by modifying the food menu. Generally, the efforts on the ground to tackle undernutrition in the general population such as integrating nutrition with agricultural sectors and international aid organizations should be extended to incarcerated people by prioritizing the vulnerable groups.

## Supporting information

S1 Appendix Data collection tool to assess undernutrition in Mizan prison institute. (DOC)

S2 Appendix Data set used to assess undernutrition in Mizan prison institute. (SAV)

## Abbreviations

AOR: Adjusted odds ratio
CI: Confidence intervals
COR: Crude odds ratio
AIDS: Acquired Immune Deficiency Disease
BMI: Body Mass Index
DDS: Dietary Diversity Score
HIV: Human Immune Deficiency Virus
TB: Tuberculosis

## References

[1] United Nations. Universal decleration of human rights [Internet]. 2015. Available from: https://www.un.org/en/udhrbook/pdf/udhr_booklet_en_web.pdf.

[2] Abera SF, Adane K. One-fourth of the prisoners are underweight in Northern Ethiopia: a cross- sectional study. BMC Public Health [Internet]. 2017;17(449):1–11. Available from: 10.1186/s12889-017-4410-9 28506311 PMC5433041

[3] Lamonaca KG. Status Of Prisoners At Three Rural Haitian Prisons. EliScholar–A Digit Platf Sch Publ Yale Heal [Internet]. 2012;(January). Available from: http://elischolar.library.yale.edu/ysphtdl/1161.

[4] Noeske J, Kuaban C, Amougou G, Piubello A, Pouillot R. Pulmonary tuberculosis in the central prison of Douala, Cameroon. East Afr Med J. 2006;83(1):25–30. 10.4314/eamj.v83i1.9357 16642747

[5] World Health Organization Regional office for Europe. Prisons and Health. Enggist Stefan, Lars Møller GG and CU, editor. 2014.

[6] Martins VJB, Florê TMMT, Santos CDL, Vieira MDFA, Sawaya AL. Long-lasting effects of undernutrition. Int J Environ Res Public Health. 2011;8:1817–46. 10.3390/ijerph8061817 21776204 PMC3137999

[7] Maleta K. Undernutrition. Malawi Med J. 2006;18(4):189–205. 27529011 PMC3345626

[8] Gould C, Tousignant B, Brian G, Mckay R, Gibson R, Bailey K, et al. Cross-sectional dietary deficiencies among a prison population in Papua New Guinea. BMC Int Heal Hum Rights [Internet]. 2013;13(21):1–7. Available from: 10.1186/1472-698X-13-21 23601963 PMC3637570

[9] Rahman A, Alam R, Islam MS, Prodhan UK. Effect of Dietary Pattern on Nutritional Status of Prisoner. IOSR J Nurs Heal Sci. 2017;6(5):50–5.

[10] Qadir M, Murad R, Qadir A, Mubeen SM. Prisoners in Karachi-A Health and Nutritional Perspective. Prisoners in Karachi–A Health and Nutritional Perspective. 2017;(March).

[11] Safarian M, Arabi M, Nematy M. Evaluation of the nutritional status using the anthropometric indices and dietary intakes in the central prison of Mashhad. 2014;3(12):266–70.

[12] KOUAME Rosine A, N’DIA Frédéric K, BLEYERE Mathieu N, OUSSOU PAY Jean-Baptiste N. Evaluation of some nutritional biomarkers of the inmates in grand-bassam prison (Côte d ‘ ivoire). J Nutr Heal Food Eng. 2018;8(5):335–8.

[13] Ravaoarisoa L, Pharlin AH, Andriamifidison NZR, Andrianasolo R, Rakotomanga J de DM, Rakotonirina J. Nutritional status of female prisoners in Antanimora prison, Madagascar. Pan Afr Med J. 2019;33(119):1–7. 10.11604/pamj.2019.33.119.18170 31489097 PMC6711675

[14] Kassa T, Alle A, Tesfu M. Assessment of Nutritional Status and Associated Factors among Prisoners Living with HIV/AIDS in Kality Prison, Addis Ababa, Ethiopia. AIDS Clin Res. 2017;8(6):1–6.

[15] Rachel KK, Kigaru DMD, Nyamota MW. Dietary intake and factors affecting food service of male prisoners living with human immunodeficiency virus at selected prisons in Kenya. Int J Nutr Metab [Internet]. 2018;10(2):6–15. Available from: http://www.academicjournals.org/IJNAM.

[16] Ethiopian Public Health Institute (EPHI) [Ethiopia] and ICF. Ethiopia Mini Demographic and Health Survey: Key Indicators. Rockville, Maryland, USA; 2019.

[17] Weldeyohannes BT. Reforming prison policy to improve women-specific health and sanitary care conditions of prisons in Ethiopia. William Mary J race, gender Soc justice [Internet]. 2018;24(1):101–26. Available from: https://scholarship.law.wm.edu/cgi/viewcontent.cgi?referer = https://www.google.com/&httpsredir=1&article=1463&context=wmjowl.

[18] African Commission on Human and Peoples’ Rights. Report of the mission of the special rapporteur on prisons and conditions of detention in Africa to the Federal Democratic Republic of Ethiopia. 2004; Available from: https://www.achpr.org/public/Document/file/English/misrep_specmec_priso_ethopia_2004_eng.pdf.

[19] Alemayehu F, Ambaw F, Gutema H. Depression and associated factors among prisoners in Bahir Dar Prison, Ethiopia. 2019;19(88):1–7. 10.1186/s12888-019-2071-1 PMC6417090 30866864

[20] World Prison Brief, Institute for Crime & Justice Policy Research. World Prison Brief Data (Prison population rate) for Ethiopia [Internet]. 2018. Available from: https://www.prisonstudies.org/sites/default/files/resources/downloads/wppl_12.pdf.

[21] FAO. Dietary Assessment: A resource guide to method selection and application in low resource settings [Internet]. 2018. Available from: http://www.fao.org/3/i9940en/I9940EN.pdf.

[22] WHO STEPS Team. Ethiopia STEPS Survey 2015. WHO STEPS chronic Dis risk factor Surveill [Internet]. 2015;1–2. Available from: https://www.who.int/ncds/surveillance/steps/Ethiopia_2015_STEPS_FactSheet.pdf.

[23] Kroenke K, Spitzer RL, Williams JB. The PHQ-9: validity of a brief depression severity measure. J Gen Intern Med. 2001;16(9):606–613. 10.1046/j.1525-1497.2001.016009606.x Available from: https://www.ncbi.nlm.nih.gov/pmc/articles/PMC1495268/pdf/jgi_01114.pdf. 11556941 PMC1495268

[24] Kennedy Gina, Ballard Terri and MarieClaude Dop Nutrition and Consumer Protection Division F and AO of the UN. Guidelines for measuring household and individual dietary diversity. 2013.

[25] World Health Organization. Global Physical Activity Questionnaire (GPAQ) Analysis Guide. 2005;1–23. Available from: https://www.who.int/ncds/surveillance/steps/resources/GPAQ_Analysis_Guide.pdf?ua=1.

[26] Fawehinmi TO, Ilomäki J, Voutilainen S, Kauhanen J. Alcohol consumption and dietary patterns: The FinDrink study. PLoS One [Internet]. 2012;7(6). Available from: https://journals.plos.org/plosone/article/file?id=10.1371/journal.pone.0038607&type=printable. 10.1371/journal.pone.0038607 22719905 PMC3373562

[27] Legesse TG, Bedane DG. Prevalence of under nutrition and associated factors among khat chewers in khat chewing shops at Gulalle Sub City, Addis Ababa, Ethiopia. J Pharm Nutr Sci [Internet]. 2016;6(4):144–52. Available from: https://pdfs.semanticscholar.org/8784/2ec7786b549d1a16e9f6671644b489731984.pdf.

[28] CDC. National Health Interview Survey. In. Available from: https://www.cdc.gov/nchs/nhis/tobacco/tobacco_glossary.htm.

[29] FAO. Hunger and food insecuriity in Ethiopia (FAOSTAT) [Internet]. 2019. Available from: http://www.fao.org/faostat/en/#country/238.

[30] Abdu Z, Kabeta T, Dube L, Tessema W, Abera M. Prevalence and associated factors of depression among prisoners in Jimma town prison, South West Ethiopia. 2018;1–10. Available from: http://downloads.hindawi.com/journals/psychiatry/2018/5762608.pdf. 10.1155/2018/5762608 PMC6029452 30018974

[31] Fiske A, Wetherell JL, Gatz M. Depression in Older Adults. Annu Rev Clin Psychol. 2009;5:363–89. 10.1146/annurev.clinpsy.032408.153621 19327033 PMC2852580

[32] Ross LJ, Wilson M, Banks M, Rezzanah F, Daglish M. Prevalence of malnutrition and nutritional risk factors in patients undergoing alcohol and drug treatment. Nutrition-Elsivier. 2012;28(7–8):738–43. 10.1016/j.nut.2011.11.003 22356728

[33] Gary S. Cuddeback, Ph.D. Anna Scheyett, Ph.D. Carrie Pettus-Davis MSW. General Medical Problems of Incarcerated Person. NIH Public Access [Internet]. 2010;1(61):45–9. Available from: https://www.ncbi.nlm.nih.gov/pmc/articles/PMC2829837/pdf/nihms177482.pdf. 10.1176/appi.ps.61.1.45 PMC2829837 20044417

[34] Holzer HJ, Stoll MA, Raphael S. Employment Dimensions of Reentry: Understanding the Nexus between Prisoner Reentry and Work. New York Univ Law Sch [Internet]. 2003; Available from: https://peerta.acf.hhs.gov/content/employment-dimensions-reentry-understanding-nexus-between-prisoner-reentry-and-work-can.

[35] Schauss A. Diet, Crime and Delinquency. Nation Crim Justice Ref Serv USA. 1980;.

[36] Penal reform International. A model for good prison farm management in africa [Internet]. 2002. 1–12 p. Available from: https://cdn.penalreform.org/wp-content/uploads/2013/06/rep-2002-prison-farm-management-en_0.pdf.

[37] Ickes SB, Wu M, Mandel MP, Roberts AC. Associations between social support, psychological well-being, decision making, empowerment, infant and young child feeding, and nutritional status in Ugandan children ages 0 to 24 months. Matern Child Nutr. 2017;14(e12483):1–11. 10.1111/mcn.12483 PMC6866247 28782300

[38] Engle PL, Ricciuti HN. Psychosocial aspects of care and nutrition. United Nations University.

[39] Potter GG, McQuoid DR, Steffens DC. Appetite Loss and Neurocognitive Deficits in Late-Life Depression. Int J Geriatr Psychiatry. 2015;30(6):647–54. 10.1002/gps.4196 25315155 PMC4691536

[40] Balafoutas L, García-gallego A, Georgantzis N, Jaber-lopez T, Mitrokostas E. Games and economic behavior rehabilitation and social behavior: Experiments in prison. Elsevier [Internet]. 2019;119(2020):148–71. Available from: 10.1016/j.geb.2019.10.009.

[41] Steger MF, Kashdan TB. Depression and everyday social activity. J Couns Psychol [Internet]. 2009;56(2):289–300. Available from: https://www.ncbi.nlm.nih.gov/pmc/articles/PMC2860146/pdf/nihms-182975.pdf. 10.1037/a0015416 20428460 PMC2860146

[42] Sharma A, Madaan V, Petty FD. Exercise for Mental Health. Prim Care Companion J Clin Psychiatry. 2006;8(2):106. 10.4088/pcc.v08n0208a 16862239 PMC1470658

[43] Ameya G, Zerdo Z, Tesfaye M, Jabesa C, Awaje A, Dejene K, et al. Intestinal parasite infections and associated factors among inmates of Arba Minch prison, southern Ethiopia: BMC Infect Dis [Internet]. 2019;19(1086):4–11. Available from: https://bmcinfectdis.biomedcentral.com/track/pdf/10.1186/s12879-019-4703-y. 10.1186/s12879-019-4703-y PMC6937967 31888496

[44] Agbesi EK. Causes and Effects of Overcrowding at Prisons: A Study at the Ho Central Prison, Ghana. Public Policy Adm Res. 2016;6(5):1–11.

[45] Karyadi E, Schultink W, Nelwan RHH, Gross R, Amin Z, Dolmans WM V., et al. Poor Micronutrient Status of Active Pulmonary Tuberculosis Patients in Indonesia. J Nutr. 2000;130(12):2953–8. 10.1093/jn/130.12.2953 11110853

[46] Stephenson LS, Latham MC, Ottesen EA. Malnutrition and parasitic helminth infections. Cambridge Univ Press Parasitol. 2001;121(SUPPL.):S23–38. 10.1017/s0031182000006491 11386688

[47] Cook Z, Kirk S, Lawrenson S, Sandford S. Use of BMI in the assessment of undernutrition in older subjects. Proc Nutr Soc. 2005;64:313–7. 10.1079/pns2005437 16048662

