# Supplementary material for: Undernutrition and associated factors among incarcerated people in Mizan prison institute, southwest Ethiopia
Source: PLoS One. 2021 May 11;16(5):e0251364. doi: 10.1371/journal.pone.0251364 (PMC8112703; doi:10.1371/journal.pone.0251364)
Supplement: S1 Appendix — (DOC) [file pone.0251364.s001.doc]

**Data collection tool to assess undernutrition in Mizan prison institute**

Note: These are both an English and Amharic version questionnaires. The Amharic version questionnaire was used to collect data.

| **S.no** | | | **Question** | | | | | | | |  | | | | | | | | | | | | | | | | **Skip** | | | | | | |
| --- | --- | --- | --- | --- | --- | --- | --- | --- | --- | --- | --- | --- | --- | --- | --- | --- | --- | --- | --- | --- | --- | --- | --- | --- | --- | --- | --- | --- | --- | --- | --- | --- | --- |
| **Part 1. Socio demographic characteristics** | | | | | | | | | | | | | | | | | | | | | | | | | | | | | | | | | |
| Q101 | | | Sex | | | | | | | | 1.Male  2.Female | | | | | | | | | | | | | | | |  | | | | | | |
| Q102 | | | Age | | | | | | | | ------------- Yrs. | | | | | | | | | | | | | | | |  | | | | | | |
| Q103 | | | Previous residence | | | | | | | | 1.Rural  2.Urban | | | | | | | | | | | | | | | |  | | | | | | |
| Q104 | | | Religion | | | | | | | | - 1. Orthodox   2. Protestant   3. Muslim   4. Catholic   5. Other specify -------- | | | | | | | | | | | | | | | |  | | | | | | |
| Q105 | | | What is your marital status? | | | | | | | | 1. Single  2. Married  3. Divorced  4. Widowed  5. Separated | | | | | | | | | | | | | | | |  | | | | | | |
| Q106 | | | What is your educational status? | | | | | | | | 1.Not read and write  2.Read & write  3.Primary (1-8)  4.Secondary (9-12)  5.Tertiary(collage/university) | | | | | | | | | | | | | | | |  | | | | | | |
| Q107 | | | Previous occupation | | | | | | | | 1. Farmer 2. Employee 3. Student 4. Housewife 5. Jobless 6. Others specify-------- | | | | | | | | | | | | | | | |  | | | | | | |
| Q108 | | | Do you have a support from family or others? | | | | | | | | 1. Yes  2. No | | | | | | | | | | | | | | | | If no skip to 201 | | | | | | |
| Q109 | | | What type of support do you get? | | | | | | | | - 1. Financial   2. Food   3. Other specify --------- | | | | | | | | | | | | | | | |  | | | | | | |
| **Part 2. Prison related factors** | | | | | | | | | | | | | | | | | | | | | | | | | | | | | | | | | |
| Q201 | | | Duration of imprisonment | | | | | | | | | ---------- months and --------years | | | | | | | | | | | | | | |  | | | | | | |
| Q202 | | | Have you ever been imprisoned previously? | | | | | | | | | 1.Yes  2.No | | | | | | | | | | | | | | |  | | | | | | |
| Q203 | | | Do you have any income-generating job in the prison? | | | | | | | | | 1.Yes  2.No | | | | | | | | | | | | | | |  | | | | | | |
| **Part 3. Nutrition related factors** | | | | | | | | | | | | | | | | | | | | | | | | | | | | | | | | | |
| Q301 | | | How frequent do you consume meal per day? | | | | | | | | | 1. Once  2. Twice  3. Three  4. Other specify------------- | | | | | | | | | | | | | | | |  | | | | | |
|  | | | Please describe the foods (meals and snacks) that you ate or drank yesterday during the day and night, whether the food is given from the prison cafeteria or other. Start with the first food or drink of the morning | | | | | | | | | | | | | | | | | | | | | | | | |  | | | | | |
|  | | | Breakfast | | Snack | | Lunch | | | | | | Snack | | | | Dinner | | | | | | | Snack | | | | | | | | | |
|  |  |  |  |  |  |  |  |  |  |  |  |  |  |  |  |  |  |  |  |  |  |  |  |  |  |  |  |  |  |  |  |  |  |
|  | | | Write down all foods and drinks mentioned. When composite dishes are mentioned, ask for the list of ingredients. When the respondent has finished, probe for meals and snacks not mentioned. | | | | | | | | | | | | | | | | | | | | | | | | |  | | | | | |
|  | | | **Food groups** | | | | | | **Examples** | | | | | | | | | | | | | | | | | | | Yes | | | No | | |
|  | | | - 1. starchy staples | | | | | | corn/maize, rice, wheat, sorghum, millet or any other grains or foods made from these (e.g. bread, noodles, porridge ) | | | | | | | | | | | | | | | | | | | 1 | | | 2 | | |
|  | | | - 1. Vitamin A- rich dark green leafy vegetables | | | | | | cassava leaves, Kale, Spinach | | | | | | | | | | | | | | | | | | | 1 | | | 2 | | |
|  | | | - 1. VitaminA rich fruits | | | | | | Mango ,Pumpkin , Carrot | | | | | | | | | | | | | | | | | | | 1 | | | 2 | | |
|  | | | - 1. Other fruits | | | | | | Banana ,oranges | | | | | | | | | | | | | | | | | | | 1 | | | 2 | | |
|  | | | - 1. Other vegetables | | | | | | Onion , potato ,Tomato | | | | | | | | | | | | | | | | | | | 1 | | | 2 | | |
|  | | | - 1. Flesh food | | | | | | Beef, pork, lamb, goat, chicken ,liver, kidney, heart or other organ meats | | | | | | | | | | | | | | | | | | | 1 | | | 2 | | |
|  | | | - 1. Eggs | | | | | | Chicken any other egg | | | | | | | | | | | | | | | | | | | 1 | | | 2 | | |
|  | | | - 1. Nuts and seeds | | | | | | Nuts, seeds | | | | | | | | | | | | | | | | | | | 1 | | | 2 | | |
|  | | | - 1. Beans and peas | | | | | | Beans, peas ,lentils | | | | | | | | | | | | | | | | | | |  | | |  | | |
|  | | | - 1. all dairy | | | | | | milk, cheese, yogurt or other milk products | | | | | | | | | | | | | | | | | | | 1 | | | 2 | | |
| Q303 | | | Do you get any additional food other than the food that is provided from the prison ? | | | | | | | | | | | | | | | | | - 1. Yes   2. 2. No | | | | | | | | If no skip to 401.1 | | | | | |
| Q304 | | | From whom you get additional food? | | | | | | | | | | | | | | | | | 1.From relative’s  2.From visitors  3.Other------- | | | | | | | |  | | | | | |
| Q305 | | | How many days do you get additional food per week? | | | | | | | | | | | | | | | | -------------------------- | | | | | | | | |  | | | | | |
| **Part 4. Physical activity**  **Q 401 Work** | | | | | | | | | | | | | | | | | | | | | | | | | | | | | | | | | |
| 401.1 | | Do you have any work in the prison? | | | | | | | | | | | | | | | | - 1. Yes   2. 2.No | | | | | | | | | | | If no skip to 402.1 | | | | |
| 401.2 | | Does your work involve vigorous-intensity activity that causes large increases in breathing or heart rate like *[carrying or lifting heavy loads, digging or construction]* for at least 10 minutes? | | | | | | | | | | | | | | | | 1.Yes  2.No | | | | | | | | | | | If no skip to 401.5 | | | | |
| 401.3 | | In a typical week, on how many days do you do vigorous intensity activities as part of your work? | | | | | | | | | | | | | | | | -------------- Days | | | | | | | | | | |  | | | | |
| 401.4 | | How much time do you spend doing vigorous-intensity activities at work on a typical day? | | | | | | | | | | | | | | | | --------- Hrs. --------- Minute | | | | | | | | | | |  | | | | |
| 401.5 | | Does your work involve moderate-intensity activity, that causes small increases in breathing or heart rate such as brisk walking *[or carrying light loads]*for at least for at least 10 minutes continuously? | | | | | | | | | | | | | | | | 1.Yes  2.No | | | | | | | | | | |  | | | | |
| 401.6 | | In a typical week, on how many days do you do moderate intensity activities as part of your work? | | | | | | | | | | | | | | | | ------------- Days | | | | | | | | | | |  | | | | |
| 401.7 | | How much time do you spend doing moderate-intensity activities at work on a typical day? | | | | | | | | | | | | | | | | --------- Hrs. --------- Minute | | | | | | | | | | |  | | | | |
| **4.2 Recreational activities** | | | | | | | | | | | | | | | | | | | | | | | | | | | | | | | | | |
| 402.1 | | | Do you do any sport or recreational activates in the prison? | | | | | | | | | | | | | | | | | | | 1. Yes  2. No | | | | | | | | | | If no skip to 501 | |
| 402.2 | | | Do you do any vigorous-intensity sports, fitness or recreational *(leisure)* activities that cause large increases in breathing or heart rate like *[running or football]* for at for at least 10 minutes continuously? | | | | | | | | | | | | | | | | | | | 1. Yes  2. No | | | | | | | | | | If no skip to 402.5 | |
| 402.3 | | | In a typical week, on how many days do you do vigorous-intensity sports, fitness or recreational *(leisure)* activities? | | | | | | | | | | | | | | | | | | | ------------- Days | | | | | | | | | |  | |
| 402.4 | | | How much time do you spend doing vigorous-intensity sports, fitness or recreational activities on a typical day? | | | | | | | | | | | | | | | | | | | ---------Hrs.-------Minute | | | | | | | | | |  | |
| 402.5 | | | Do you do any moderate-intensity sports, fitness or recreational (leisure) activities that cause a small increase in breathing or heart rate such as brisk walking, [cycling, and volleyball] for at least 10 minutes continuously? | | | | | | | | | | | | | | | | | | | 1. Yes  2. No | | | | | | | | | |  | |
| 402.6 | | | In a typical week, on how many days do you do moderate-intensity sports, fitness or recreational *(leisure)* activities? | | | | | | | | | | | | | | | | | | | ------------- Days | | | | | | | | | |  | |
| 402.7 | | | How much time do you spend doing moderate-intensity sports, fitness or recreational *(leisure)* activities on a typical day? | | | | | | | | | | | | | | | | | | | ---------Hrs.-------Minute | | | | | | | | | |  | |
| **Part 5. Medical factors** | | | | | | | | | | | | | | | | | | | | | | | | | | | | | | | | | |
| Q501 | | | Had you been treated for TB within the past 12 months? | | | | | | | | | | | | 1.Yes  2.No | | | | | | | | | | |  | | | | | | | |
| Q502 | | | Had you been treated for malaria in the past 2 weeks ? | | | | | | | | | | | | 1.Yes  2.No | | | | | | | | | | |  | | | | | | | |
| Q503 | | | Had you been treated for diarrhea in the past 2 weeks ? | | | | | | | | | | | | 1.Yes  2.No | | | | | | | | | | |  | | | | | | | |
| Q504 | | | Had you ever been tested for HIV? | | | | | | | | | | | | 1.Yes  2. No | | | | | | | | | | | If no skip to 507 | | | | | | | |
| Q505 | | | What was your test result? | | | | | | | | | | | | 1. HIV negative  2. HIV positive | | | | | | | | | | |  | | | | | | | |
| **Depression** | | | | | | | | | | | | | | | | | | | | | | | | | | | | | | | | | |
| Q506 | Depression | | | Patient health questionnaire (PHQ-9) | | | | | | | | | | | | | | | | | Not at all | | Several  days | | More than half the Days | | | | | | | | Nearly  every day |
|  |  |  |  | 1.Little interest or pleasure in doing things | | | | | | | | | | | | | | | | |  | |  | |  | | | | | | | |  |
|  |  |  |  | 2.Feeling down, depressed, or hopeless | | | | | | | | | | | | | | | | |  | |  | |  | | | | | | | |  |
|  |  |  |  | 3.Trouble falling or staying asleep, or sleeping too much | | | | | | | | | | | | | | | | |  | |  | |  | | | | | | | |  |
|  |  |  |  | 4.Feeling tired or having little energy | | | | | | | | | | | | | | | | |  | |  | |  | | | | | | | |  |
|  |  |  |  | 5.Poor appetite or overeating | | | | | | | | | | | | | | | | |  | |  | |  | | | | | | | |  |
|  |  |  |  | 6.Feeling bad about yourself or that you are a failure or have let yourself or your family down | | | | | | | | | | | | | | | | |  | |  | |  | | | | | | | |  |
|  |  |  |  | 7.Trouble concentrating on things, such as reading the newspaper or watching television | | | | | | | | | | | | | | | | |  | |  | |  | | | | | | | |  |
|  |  |  |  | 8. Moving or speaking so slowly that other people could have noticed. Or the opposite being so fidgety or restless that you have been moving around a lot more than usual | | | | | | | | | | | | | | | | |  | |  | |  | | | | | | | |  |
|  |  |  |  | 9.Thoughts that you would be better off dead, or of  hurting yourself | | | | | | | | | | | | | | | | |  | |  | |  | | | | | | | |  |
| **Part 6. Behavior related factors** | | | | | | | | | | | | | | | | | | | | | | | | | | | | | | | | | |
| Q601 | | | Had you ever smoked cigarettes? | | | | | | | | | | | 1. Yes 2. No | | | | | | | | | | | | | | | | If no skip to 605 | | | |
| Q602 | | | For how long did you smoke cigarette before you entered to this prison? | | | | | | | | | | | ----------- Days--------- Months -------Years | | | | | | | | | | | | | | | |  | | | |
| Q603 | | | For how many days did you smoke per week? | | | | | | | | | | | - 1. Dailey   2. Once  3. Twice  4. Three  5. Four  6. Five  7. Six | | | | | | | | | | | | | | | |  | | | |
| Q604 | | | How many cigarettes did you smoke per day? | | | | | | | | | | | ------------------------- | | | | | | | | | | | | | | | |  | | | |
| Q605 | | | Had you ever chewed Khat? | | | | | | | | | | | 1. Yes 2. No | | | | | | | | | | | | | | | | If no skip to 609 | | | |
| Q606 | | | For how long did you chew Khat before you entered to this prison? | | | | | | | | | | | ------------ Days--------- Months -------Years | | | | | | | | | | | | | | | |  | | | |
| Q607 | | | For how many days did you chew Khat per week? | | | | | | | | | | | ------------------- Days | | | | | | | | | | | | | | | |  | | | |
| Q608 | | | For how long do you chew per session of chewing Khat? | | | | | | | | | | | --------- Hrs. --------- Minute | | | | | | | | | | | | | | | |  | | | |
| Q609 | | | Have you ever consumed any alcohol such as beer, wine, tela, teje ? | | | | | | | | | | | - - - 1. Yes       2. No | | | | | | | | | | | | | | | | If no skip to 701 | | | |
| Q6010 | | | Which drink do you mostly drink? | | | | | | | | | | | - - 1. Beer     2. Tela     3. Teje     4. Areqe     5. Wine     6. Other ------------------ | | | | | | | | | | | | | | | |  | | | |
| Q6011 | | | How often did you drink standard alcoholic drink per week? | | | | | | | | | | | ------------------- | | | | | | | | | | | | | | | |  | | | |
| Q6012 | | | How much standard alcoholic drink did you drunk on a typical day of the week? | | | | | | | | | | | ------------------- | | | | | | | | | | | | | | | |  | | | |
| **Part 7. Environmental factors** | | | | | | | | | | | | | | | | | | | | | | | | | | | | | | | | | |
| Q701 | | | How is your sleeping condition? | | | | | 1.In group  2.Individually | | | | | | | | | | | | | | | | | | | | | |  | | | |
| **Part 8. Anthropometric measurements** | | | | | | | | | | | | | | | | | | | | | | | | | | | | | | | | | |
| **Measurement** | | | | | | **1** | | | | **2** | | | | | | **Average** | | | | | | | | | | | | | | | | | |
| Q801 | | | Weight in kilogram | | |  | | | |  | | | | | |  | | | | | | | | | | | | | | | | | |
| Q802 | | | Height in meter | | |  | | | |  | | | | | |  | | | | | | | | | | | | | | | | | |

Data collector’s name ______________________signature ________date______

Supervisor’s name ______________________signature ________date________

**Thank you very much!!**

| **ተ.ቁ** | **ጥያቄ**  **Amharic version questionnaire** | | | | | መልስ | | | | | | | | | | | | | | **ዝለል** | |
| --- | --- | --- | --- | --- | --- | --- | --- | --- | --- | --- | --- | --- | --- | --- | --- | --- | --- | --- | --- | --- | --- |
| **ክፍል 1. ማኅበራዊና ሥነ ሕዝባዊ ሁኔታዎች** | | | | | | | | | | | | | | | | | | | | | |
| 101 | | | ጾታ | | | 1.ወንድ  2.ሴት | | | | | | | | | | | | | |  | |
| 102 | | | ዕድሜ | | | ________________ዓመት. | | | | | | | | | | | | | |  | |
| 103 | | | የቀድሞ መኖሪያ | | | 1.ገጠር  2.ከተማ | | | | | | | | | | | | | |  | |
| 104 | | | ሃይማኖት | | | 1. ኦርቶዶክስ  2. ፕሮቴስታንት  3. ሙስሊም  4. ካቶሊክ  5. ሌላ ይጠቀስ ____________________ | | | | | | | | | | | | | |  | |
| 105 | | | የጋብቻ ሁኔታ | | | 1. ያላገባ  2. ያገባ  3. የተፋታ  4. የሞተባት /የሞተችበት/  5. ተለያይተው የሚኖሩ | | | | | | | | | | | | | |  | |
| 106 | | | የትምህርት ደረጃ | | | 1. ማንበብና መጻፍ የማይችል  2. ማንበብና መጻፍ የሚችል  3. 1ኛ ደረጃ (1-8)  4. 2ኛ ደረጃ (9-12)  5. 3ኛ ደረጃ (ኮሌጅ / ዩኒቨርስቲ) | | | | | | | | | | | | | |  | |
| 107 | | | የቀድሞ ሥራ | | | 1. አርሶ አደር 2. የመንግስት / የግል ተቁዋም ሠራተኛ 3. ተማሪ 4. የቤት እመቤት 5. ሥራ የሌለው 6. ሌላ ካለ ይገለጽ____________________ | | | | | | | | | | | | | |  | |
| 108 | | | ከቤተሰብ ወይም ከሌሎች ድጋፍ አሎት? | | | 1. አዎን  2. አይደለም | | | | | | | | | | | | | | መልሶ.አይደለመ ከሆነወደ#**201** ይሂዱ | |
| 109 | | | ምን አይነት ድጋፍ ያገኛሉ? | | | 1.ገንዘብ  2. ምግብ  3. ሌላ ይጠቀስ ____________________ | | | | | | | | | | | | | |  | |
| **ክፍል 2. ከማረሚያ ተቋም ጋር የተገናኙ ጉዳዮች** | | | | | | | | | | | | | | | | | | | | | |
| 201 | | | በዚህ ማረሚያ ተቋም ምን ያህል ጊዜ ቆይተዋል? | | | | | _______ወር_______ዓመት | | | | | | | | | | | |  | |
| 202 | | | ከዚህ ቀደም ታስረው ያውቃሉ? | | | | | 1. አዎን  2. አይደለም | | | | | | | | | | | |  | |
| 203 | | | በዚህ ማረሚያ ውስጥ ገቢ የሚያገኙበት ሥራ አለዎት? | | | | | 1. አዎን  2. አይደለም | | | | | | | | | | | |  | |
| **ክፍል 3. ከሥነ ምግብ ጋር የተያያዙ ጉዳዮች** | | | | | | | | | | | | | | | | | | | | | |
| 301 | | | ባለፉት 4 ሳምንታት ውስጥ ባብዛኛው በቀን ስንት ጊዜ ምግብ ይመገባሉ? | | | | | 1. አንዴ  2. ሁለቴ  3. ሶስቴ  4. ሌላ (ይጠቀስ)__________ | | | | | | | | | | | |  | |
| 302 | | | ባለፉት 24 ስዓታት ውስጥ ምን ተመግበዋል ?  ቁርስ ---------------------------------------------------------------- ተጨማሪ ምግብ --------------------------------------------------  ምሳ --------------------------------------------------------------- ተጨማሪ ምግብ ----------------------------------------------------  እራት ---------------------------------------------------------------- ተጨማሪ ምግብ --------------------------------------------------- | | | | | | | | | | | | | | | | | | |
| ባለፉት 24 ስዓታት ውስጥ ከተዘረዘሩት የምግብ አይነቶች ውስጥ ስንቱን ተመግበዋል ? | | | | | | | | | | | | | | | | | | | | | |
| **የምግብ አይነቶች** | | | | | | | | **ምሳሌዎች** | | | | | | | | | | | 1.አዎ 2.አይደለም | | |
| 1. እህል እና ጥራጥሬ | | | | | | | | የበቆሎ፣ ጤፍ ፣ማሽላ፣ስንዴ፣ሩዝ፣ ወይም ከእነዚህ  ዳቦዎች፣ፓስታ፣ ሞኮሮኒ  ወዘተ | | | | | | | | | | | | | 1. 2. |
| 1. በቫይታሚን ኤ የበለፀጉ ባለጥቁር አረንጓዴ ቅጠል አትክልቶች ; | | | | | | | | ጎመን ፣ ጎደሬ | | | | | | | | | | | | | 1. 2. |
| 1. በቫይታሚን ኤ የበለፀጉ ፍራፍሬዎች | | | | | | | | ማንጎ፣የበሰለ ፓፓያ፣ዱባ፣ካሮት | | | | | | | | | | | | | 1. 2. |
| 1. ሌሎች ፍራፍሬዎች | | | | | | | | ሙዝ፣ ብርቱካን | | | | | | | | | | | | | 1. 2. |
| 1. ሌሎች አትክልቶች | | | | | | | | ቲማቲም፣ሽንኩርት | | | | | | | | | | | | |  |
| 1. ሥጋ እና ዓሳ | | | | | | | | የበሬ ሥጋ፣ በግ፣ ፍየል፣ ዶሮ፣ ጉበት፣ ኩላሊት፣ ልብ፣ በደም ላይ የተመሠረቱ ምግቦች | | | | | | | | | | | | | 1. 2. |
| 1. እንቁላል | | | | | | | | የዶሮ ወይንም የሌላ እንቁላል | | | | | | | | | | | | | 1. 2. |
| 1. ለውዝ እና ዘሮች | | | | | | | | ለውዝ፣ ሰሊጥ | | | | | | | | | | | | | 1. 2. |
| 1. ባቄላ፣አተር | | | | | | | | ባቄላ፣አተር፣ምስር | | | | | | | | | | | | |  |
| 1. ወተት እና የወተት ምርቶች | | | | | | | | ወተት፣አይብ፣እርጎወይምሌሎችየወተትተዋጽኦዎች | | | | | | | | | | | | | 1. 2. |
| 303 | | በማረሚያ ቤቱ ከሚሰጠው ምግብ በተጨማሪ ሌላ ምግብ ይመገባሉ? | | | | | | | 1.አዎን  2. አይደለም | | | | | | | | | መልሱ አይደለም ከሆነ ወደ **#401.1** ይሂዱ | | | |
| 304 | | ተጨማሪምግቡን ከየት ያገኛሉ? | | | | | | | 1.በግዥ  2. ከዘመድ  3. ከሌሎች ጎብኝዎች  4. ሌላ _________________ | | | | | | | | |  | | | |
| 305 | | በሳምንት ምን ያህል ጊዜ ተጨማሪ ምግብ ይመገባሉ? | | | | | | | **_________________** | | | | | | | | |  | | | |
| **ክፍል 4. የሰውነት እንቅስቃሴ (401 ከስራ ጋር የተያያዘ )** | | | | | | | | | | | | | |  | | | | | |  | |
| 401.1 | | | | በዚህ ማረሚያ ውስጥ ሥራ አልዎት? | | | | | | | | | | 1. አዎን  2. አይደለም | | | | | | መልሱ አይደለም ከሆነ ወደ **#402.1** ይሂዱ | |
| 401.2 | | | | ሥራዎት በአብዛኛው ከባድ ሆኖ( ለምሳሌ ከባድ ሸክም ማንሳት፣ መቆፈር ወይንም ግንባታ) ትንፋሽዎንና የልብ ትርታዎን ቢያንስ ለ10 ደቂቃ የሚጨምር ነው? | | | | | | | | | | 1. አዎን  2. አይደለም | | | | | | መልሱ አይደለም ከሆነ ወደ #**401.5** ይሂዱ | |
| 401.3 | | | | በአብዛኛው በሳምንት ለምን ያህል ቀናት ከባባድ ሥራዎችንና እንቅስቃሴዎችን ይሰራሉ? | | | | | | | | | | ___________ ቀን | | | | | |  | |
| 401.4 | | | | በአንድ ቀን ከባባድ ሥራዎችንና እንቅስቃሴዎችን በማድረግ ምን ያህል ጊዜ ይቆያሉ? | | | | | | | | | | ­­­________ሰዓት ________ደቂቃ | | | | | |  | |
| 401.5 | | | | ሥራዎት በአብዛኛውመጠነኛ ክብደት ያለው ሆኖ ( ለምሳሌ ፈጠን ያለ እርምጃ ፣ ቀላል ሸክም ማንሳት) ትንፋሽዎንና የልብ ትርታዎን በትንሹ ቢያንስ ለ10 ደቂቃ የሚጨምር ነው? | | | | | | | | | | 1. አዎን  2. አይደለም | | | | | |  | |
| 401.6 | | | | በአብዛኛው በሳምንት መጠነኛ ሥራዎችንና እንቅስቃሴዎችን ለምን ያህል ቀናት ይሰራሉ? | | | | | | | | | | ___________ ቀን | | | | | |  | |
| 401.7 | | | | በአንድ ቀን መጠነኛ ሥራዎችንና እንቅስቃሴዎችን በማድረግ ምን ያህል ጊዜ ይቆያሉ? | | | | | | | | | | ­­­________ሰዓት ________ደቂቃ | | | | | |  | |
| **4.2 የመዝናኛ እንቅስቃሴዎች** | | | | | | | | | | | | | | | | | | | | | |
| **4**02.1 | | | | በማረሚያ ቤት ውስጥ ስፖርታዊ እንቅስቃሴዎችን ወይም ጨዋታዎችን ያደርጋሉ ? | | | | | | | | | 1. አዎን  2. አይደለም | | | | | | | መልሱ አይደለምከሆነ ወደ #**501**ይሂዱ | |
| **4**02.2 | | | | በአብዛኛውከባባድ ስፖርታዊ እንቅስቃሴዎችን ወይም ጨዋታዎች እንደ ( ሩጫ ፤ እግር ኳስጨዋታ ) ትንፋሽዎንወይም የልብ ትርታዎን ቢያንስ ለ10 ደቂቃ የሚጨምር ይሰራሉ? | | | | | | | | | 1. አዎን  2. አይደለም | | | | | | | መልሱ አይደለም ከሆነ ወደ #**402.5** ይሂዱ | |
| **4**02.3 | | | | በአብዛኛው በሳምንት ከባባድ ስፖርታዊ እንቅስቃሴዎችን ወይም ጨዋታዎች ለምን ያህል ቀናት ይሰራሉ? | | | | | | | | | ___________ ቀን | | | | | | |  | |
| **4**02.4 | | | | ከባባድ ስፖርታዊ እንቅስቃሴዎችን ወይም ጨዋታዎች በሰሩ ዕለት ለምን ያህል ጊዜ ይሰራሉ? | | | | | | | | | ­­­  _____ሰዓት_______ደቂቃ | | | | | | |  | |
| **4**02.5 | | | | በአብዛኛውመጠነኛ ስፖርታዊ እንቅስቃሴዎችን ወይም ጨዋታዎች እንደ ( የእጅ ኳስጨዋታ፣ ፈጠን ያለ እርምጃ) ያሉ የልብ ትርታን ወይምትንፋሽ በትንሹ ቢያንስ ለ10 ደቂቃ የሚጨምሩ ይሰራሉ? | | | | | | | | | 1. አዎን  2. አይደለም | | | | | | |  | |
| **4**02.6 | | | | በሳምንት ለምን ያህል ጊዜ መጠነኛ ስፖርታዊ እንቅስቃሴዎችን (ጨዋታዎችን) ይሰራሉ? | | | | | | | | | ___________ ቀን | | | | | | |  | |
| **4**02.7 | | | | መጠነኛስፖርታዊ እንቅስቃሴዎችን (ጨዋታዎችን) በሰሩ ዕለት ለምን ያህል ጊዜ ይሰራሉ? | | | | | | | | | ____ሰዓት_____ ደቂቃ | | | | | | |  | |
| **ክፍል 5. የሕክምና ጉዳዮች** | | | | | | | | | | | | | | | | | | | | | |
| 501 | | | | ባለፉት 12 ወራት ለሳንባ ነቀርሳ (TB) ታክመው ያውቃሉ? | | | | | | | 1. አዎን  2. አይደለም | | | | | | | | |  | |
| 502 | | | | ባለፉት 2 ሳምንታት ለወባ ታክመው ያውቃሉ? | | | | | | | 1. አዎን  2. አይደለም | | | | | | | | |  | |
| 503 | | | | ባለፉት 2 ሳምንታት ለተቅማጥ ታክመው ያውቃሉ? | | | | | | | 1. አዎን  2. አይደለም | | | | | | | | |  | |
| 504 | | | | ባለፉት 6 ወራት ፀረ- ትላትል መድሃኒት ወስደው ያውቃሉ? | | | | | | | 1. አዎን  2. አይደለም | | | | | | | | |  | |
| 505 | | | | የHIV ምርመራ አድርገው ያውቃሉ? | | | | | | | 1. አዎን  2. አላውቅም | | | | | | | | | መልሱ አላውቅም ከሆነ ወደ #**507** ይሂዱ | |
| 506 | | | | የምርመራ ውጤትዎ ምን ነበር ? | | | | | | | 1.የለም  2. አለ | | | | | | | | |  | |
| **507 ስለ ድብርት ( የታካሚ ጤንነት መጠይቅ (PHQ-9))**  **ማሳሰቢያ** - የደመሩት ውጤት ከ15 በላይ ከሆነ እና በጥያቄ ተራ ቁጥር 9 የመረጡት መልስ 1፣2 ወይም 3 ከሆነ ተሳታፊው የህክምና እርዳታ ስለሚያስፈልጋቸው ለበለጠ ምርመራ ወደ ተመላላሽ ክፍል (OPD ) ይላኩ | | | | | | | | | | | | | | | | | | | | | |
| **ባለፉት 2 ሳምንታት ውስጥ የሚከተሉትን ተግባራት ለምን ያህል ጊዜ እንደ ፈፀሙ በ ☑ ያመልክቱ** | | | | | | | | | | **በፍጹም** | | **አንዳንድ ቀን** | | | | | **ከግማሽ ቀን በላይ** | | **በየቀኑማለት ይቻላል** | | |
| 1. ሥራዎችን ለመስራት አነስተኛ ፍላጎት | | | | | | | | | | 0 | | 1 | | | | | 2 | | 3 | | |
| 2. መከፋት፣ መደበር ወይንም ተስፋ መቁረጥ | | | | | | | | | | 0 | | 1 | | | | | 2 | | 3 | | |
| 3.ለመተኛት መቸገር ፣ ረዥም ሰዓት መተኛት | | | | | | | | | | 0 | | 1 | | | | | 2 | | 3 | | |
| 4. የድካም ስሜት መሰማት ወይንም የሰውነት መዛል | | | | | | | | | | 0 | | 1 | | | | | 2 | | 3 | | |
| 5.የምግብ ፍላጎት መቀነስ ወይንም አብዝቶ መመገብ | | | | | | | | | | 0 | | 1 | | | | | 2 | | 3 | | |
| 6.ራስን መጥላት ወይንም ራስን ወይም ቤተሰብን እንዳዋረዱ መሰማት | | | | | | | | | | 0 | | 1 | | | | | 2 | | 3 | | |
| 7. ሲያነቡ፣ ተሌቭዢን ሲመለከቱ ወይም ሌላ ስራ ሲሰሩ ትኩረት ለማድረግ መቸገር | | | | | | | | | | 0 | | 1 | | | | | 2 | | 3 | | |
| 8. በቀስታ ማውራት፣ መንቀሳቀስ ለሌላ ሰው እስክታወቅ ድረስ። ወይም በተቃራኒው ከተለመደው ውጭ መንቀዥቀዥ፣ እረፍት ማጣት | | | | | | | | | | 0 | | 1 | | | | | 2 | | 3 | | |
| 9. ብሞት ይሻል ነበር ብሎ ማሰብ፣ ራስን ለመጉዳት ማሰብ | | | | | | | | | | **0** | | **1** | | | | | **2** | | **3** | | |
| *ድምር* | | | | | | | | | |  | | | | | | | | | | | |
| **ክፍል 6. ከጠባይ ጋር የተያያዙ ጉዳዮች** | | | | | | | | | | | | | | | | | | | | | |
| 601 | | | | ማረሚያ ቤት ከመግባትዎ በፊት ስጋራ አጪሰው ያውቃሉ? | | | | 1. አውቃለሁ   2. አላውቅም | | | | | | | | መልሱ አላውቅም ከሆነ ወደ **#605** ይሂዱ | | | | | |
| 602 | | | | ማረሚያ ቤት ከመግባትዎ በፊት ለምን ያህል ጊዜ ስጋራ አጪሰዋል? | | | | ________ ቀን _________ ወር _________ዓመት | | | | | | | |  | | | | | |
| 603 | | | | ማረሚያ ቤት ከመግባትዎ በፊት በአብዛኛው በሳምንት ስንት ቀን ስጋራ ያጨሱ ነበር? | | | | 1. ሁሌ 2. አንዴ 3. ሁለቴ 4. ሶስቴ 5. አራቴ 6. አምስቴ 7. ስድስቴ | | | | | | | |  | | | | | |
| 604 | | | | ማረሚያ ቤት ከመግባትዎ በፊት በአብዛኛው ምን ያህል ስጋራ በቀን ያጨሱ ነበር? | | | | **_________________** | | | | | | | |  | | | | | |
| 605 | | | | ጫት ቅመው ያውቃሉ? | | | | 1.አውቃለሁ  2. አላውቅም | | | | | | | | መልሱ አላውቅም ከሆነ ወደ**#609**ይሂዱ | | | | | |
| 606 | | | | ማረሚያ ቤት ከመግባትዎ በፊት ለምን ያህል ጊዜ ጫት ቅመዋል? | | | | ________ ቀን _________ ወር _________ዓመት | | | | | | | |  | | | | | |
| 607 | | | | በሳምንት ለምን ያህል ቀን ይቅሙ ነበር? | | | | **_____________**ቀን | | | | | | | |  | | | | | |
| 608 | | | | በአብዛኛው አንድ የመቃሚያ ክፍለ ጊዜ ለምን ያህል ሰዓት ይቆይ ነበር? | | | | **_________________**ሰዓት | | | | | | | |  | | | | | |
| 609 | | | | ማረሚያ ቤት ከመግባቶ በፊት የአልኮል መጠጦችን እንደ ቢራ ፣ ወይን ፣ ጠላ ፣ጠጅ ወዘተ … ጠጥተው ያውቃሉ? | | | | 1.አውቃለሁ  2. አላውቅም | | | | | | | | መልሱ አላውቅም ከሆነ ወደ**#701**ይሂዱ | | | | | |
| 6010 | | | | በአብዛኛው ይጠጡት የነበረው መጠጥ ምን ነበር ? | | | | 1. ቢራ 2. ጠላ 3. ጠጅ 4. አረቄ 5. ወይን 6. ሌላ**________________** | | | | | | | |  | | | | | |
| 6011 | | | | በአብዛኛው በሳምንት ምን ያህል ቀን የአልኮል መጠጥ ይጠጡ ነበር? | | | | **_________________**ቀን | | | | | | | |  | | | | | |
| 6012 | | | | በአብዛኛው በቀን ምን ያህል መለኪያ ፣ ጠርሙስ ወይም ብርሌ የአልኮል መጠጥ ይጠጡ ነበር? | | | | ________ መለኪያ /________ጠርሙስ/  /_______ ኩባያ/________ ብርሌ | | | | | | | |  | | | | | |
| **ክፍል 7. ከባቢያዊ ጉዳዮች** | | | | | | | | | | | | | | | | | | | | | |
| 701 | | | | የመኝታ ሁኔታዎ እንዴት ነው? | | | 1.በቡድን 2. ለየብቻ | | | | | | | | | | | | | | |
| **ክፍል 8. የተክለሰውነትልኬቶች** | | | | | | | | | | | | | | | | | | | | | |
| **ልኬት** | | | | | **1** | | | | | | | | | | **2** | | | | | **አማካይ** | |
| 801 | | | | ክብደት በኪ.ግ |  | | | | | | | | | |  | | | | |  | |
| 802 | | | | ቁመት በሴንቲ ሜትር |  | | | | | | | | | |  | | | | |  | |

ስለተሳተፉ እጅግ በጣም አመሰግናለሁ።

የመረጃ ሰብሳቢውስም__________________ፊርማ______ቀን ____________________

የተቆጣጣሪውስም_____________________ፊርማ______ቀን ____________________
